# Effect of different planting areas on the chemical compositions and hypoglycemic and antioxidant activities of mulberry leaf extracts in Southern China

**DOI:** 10.1371/journal.pone.0198072

**Published:** 2018-06-26

**Authors:** Jing-Yi Hao, Yi Wan, Xiao-Hui Yao, Wei-Guo Zhao, Run-Ze Hu, Cong Chen, Long Li, Dong-Yang Zhang, Guo-Hua Wu

**Affiliations:** 1 College of Biotechnology and Sericultural Research Institute, Jiangsu University of Science and Technology, Zhenjiang, P.R. China; 2 Laboratory of Quality and Safty Risk Assessment for Sericulture Products and Edible Insect (Zhenjiang), Ministry of Agriculture, Zhenjiang, P.R. China; 3 Quality Inspection Center for Sericultutal Products Ministry of Agriculture, Zhenjiang, P.R. China; College of Agricultural Sciences, UNITED STATES

## Abstract

Guangdong, Guangxi and Chongqing are emerging sericulture areas in China where the production of mulberry leaves is huge. In order to identity high quality mulberry leaves that are suitable for healthy products to expand planting, 24 samples from three regions (Guangdong, Guangxi, Chongqing) in the south of China were quantified for two alkaloids (1-deoxynojirimycin and fagomine) and five phenols (chlorogenic acid, rutin, isoquercitrin, etc.) using high-performance liquid chromatography tandem mass spectrometry (HPLC-MS/MS). Additionally, the total phenolic and total flavonoid contents, antioxidant and glycosidase inhibitory activities (hypoglycemic activity) were tested using different assays (DPPH, ABTS, FRAP) to comprehensively evaluate the quality of the mulberry leaves. The contents of DNJ and fagomine ranged from 0.401±0.003 to 5.309±0.036 mg/g and from 0.279±0.031 to 2.300±0.060 mg/g, respectively. The main phenolic constituents were chlorogenic acid, rutin and isoquercitrin, with chlorogenic acid present in the highest concentrations, ranging from 3.104±0.191 to 10.050±0.143 mg/g. The antioxidant activity exhibited a tendency as follows: Guangxi > Guangdong > Chongqing, except for two samples from Chongqing, which showed the highest antioxidant activity. Based on our study, mulberry leaves from Guangdong and Guangxi could be future sources of natural hypoglycemic and antioxidant products.

## Introduction

Mulberry (*Morus alba* L.) is classified as a broad-leaved plant that has been distributed widely in China since ancient times. It can adapt to many environmental conditions ranging from the frigid zone to the tropical zone [1]. It is well known that mulberry leaves have been mainly used for feeding silkworms [2]. However, mulberry leaves contain a variety of functional components, thus, they are also a traditional Chinese medicine [3, 4]. There are many reports regarding the use of mulberry leaves for decreasing blood glucose [5, 6], inhibiting the development of atherosclerosis [7, 8], reducing obesity [9], and for their anti-oxidant [10, 11], antibacterial [12, 13], antihypertensive [14], antineoplastic [15], anti-inflammatory activities [16]. Additionally, mulberry leaves have also started to attract more attention as a plant material that can be made into mulberry-leaf tea, noodles, bean curd, mulberry-leaf wine, mulberry-leaf vinegar, etc. for their beneficial health properties [4, 17, 18].

According to previous researchers, the main active compounds of mulberry leaves are alkaloids (1-deoxynojirimycin and fagomine) [18] and phenols (flavonoids and organic acids) [10]. 1-deoxynojirimycin (DNJ) and fagomine are the most recognized biological active constituents in mulberry leaves due to their ability to decrease blood glucose. Both DNJ and fagomine exhibit the α-glycosidase inhibitory activity via competitive binding with α-glycosidase, thus inhibiting the decomposition of disaccharides into glucose [19]. Fagomine also has the ability to induce insulin secretion [20]. Moreover, phenols are also important functional components of mulberry leaves. The content of flavonoid, which is the main constituents in phenols, ranges from 9.84 to 29.60 mg/g in mulberry leaves. The flavonoids play a leading role in the aspect of antioxidant activity by directly scavenging O_2_^-^ and ·OH by single electrons [21]. As a natural antioxidant, flavonoids are also related to anti-aging and cancer prevention. Chlorogenic acid, the main organic acid in mulberry leaves, exhibits antimutagenic, anti-inflammatory and antioxidant activities [22]. Therefore, mulberry leaf is a plant material that contains a variety of functional components and is beneficial to health. Consequently, it is worthwhile to perform a thorough study on mulberry leaves.

The quality of mulberry leaves can be influenced by many environmental factors such as the cultivated varieties, geographical position, temperature, and precipitation. According to a survey, the mulberry field area of China has been estimated to be 700 thousand hectares, which brings a huge production of 15–37 tons mulberry leaves per hectare [17]. Therefore, it is necessary to make an evaluation of the quality of mulberry leaves in different areas and for different cultivars. Since 2000, the sericulture industry center of China has gradually moved westward and southward. Therefore, mulberry trees of different varieties have been widely cultivated in Guangdong, Guangxi and Chongqing. The purpose of this study is to identity high quality mulberry leaves that are suitable for healthy products to expand planting. Based on the above discussion, 24 samples from south China (Guangdong, Guangxi and Chongqing) were selected to determine the quality. These results will provide new developments for mulberry food industry and help us make full use of mulberry leaves.

## Materials and methods

### Chemicals and solvents

Standard isoquercitrin, rutin, DNJ, and fagomine were purchased from Spring and Autumn Co (Nanjing, China). DPPH (2,2-diphenyl-1-picrylhydrazyl), formic acid (HPLC, ≥98%), standard chlorogenic acid, kaempferide, and quercetin were obtained from Aladdin (Shanghai, China). All of the standards were ≥98% purity. The Folin-Ciocalteu reagent was from Sigma-Aldrich (USA), and α-glucosidase and PNPG (4-nitrophenyl-α-D-glucopyranoside) were from Baomanbio Co (Shanghai, China). Two different T-AOC assay kits (the total antioxidant capacity assay kit with the ABTS assay and the FRAP method) were obtained from Beyotime Institute of Biotechnology (Shanghai, China). Acetonitrile and methyl alcohol were of chromatographic grade (Tedia Co, USA). Water was prepared using a Milli-Q purification system (Millipore, MA, USA).

Stock solutions of each individual standard chemical were prepared in MeOH at concentrations of 1 mg/mL and stored at -4 °C. All solutions prepared for HPLC-MS/MS were filtered through 0.22-μm nylon membranes before use.

### Sample preparation

Mulberry leaf samples were collected from trees without fruits between October 2016 and November 2016 from 3 regions (Guangdong, Guangxi, Chongqing) in the south of China, and the location information and climate information are shown in S1 Fig. and Table 1, respectively. All the locations gave permission to conduct the study on the site and the field studies did not involve endangered or protected species. The leaves were naturally dried at room temperature in mulberry orchards before couriered to laboratory to prevent the destruction of the active ingredients in a high-temperature environment. Finally, the dried leaves were pulverized and kept sealed at -20 °C.

**Table 1:** Climates of the different collection areas.

No.	Origin	Latitude and Longitude	Temperature (°C)^a^	Precipitation (mm)^b^
1	Guangdong	N 20°13′~25°31′; E 109°39′~117°19′	23	131.05
2	Guangxi	N 20°54′~26°24′; E 104°26′~112°04′	23	109.72
3	Chongqing	N 28°10'~32°13'; E 105°11'~110°11'	17	110.25

^a^ Temperature means average temperature from 2016.10 to 2016.11

^b^ Precipitation means total precipitation of 2016.10 to 2016.11

### Extractions of mulberry leaves

The dried leaf powders (0.2 g) were each extracted twice with 70% ethanol (20 mL). During this process, the mixtures were sonicated in an ultrasonic-bath (KH-700DE, Kunshan Ultrasonic Instrument Co, Jiangsu, China) for 20 minutes each. After cooling to the room temperature, all of the extractions were mixed in a 50-mL volumetric flask and adjusted to the final line using 70% ethanol. The extractions were set aside in a refrigerator at -4 °C to prevent the oxidation of the active ingredients.

### Determination of total phenolic and total flavonoid contents

#### Total phenolic contents

Total phenolic contents were determined according to Zhang [23] via the Folin-Ciocalteu method. The Folin-Ciocalteu reagent was diluted with deionized water 10 times and then mixed with 40-μL extracts of each sample. The mixture was mixed with 1.2 mL of Na_2_CO_3_ solution (7.5 wt/v) after 5min of storage at room temperature. Finally, the absorbance at 765 nm was measured using a spectrophotometer after 1–2 h of reaction in a dark environment. Gallic acid was used as the control to express the total phenolic contents (mg GAE/g extract).

#### Total flavonoid contents

The total flavonoid contents were determined using the aluminum nitrate method according to previous study with slight modifications [24]. This method is based on the formation of a flavonoid-aluminum complex, which has an absorptivity maximum wavelength at 510 nm. Approximately 1 mL of extract was mixed with 0.3 mL 5% NaNO_2_ for 6 min. Then, 0.3 mL of Al(NO_3_)_3_ (5% wt/v) solution was added into the mixture and kept at room temperature for 6 min. After reaction with NaOH (4% wt/v, 4 mL) for 15 min, the mixture was adjusted to the final line with deionized water, and the absorbance at 510 nm was measured using the spectrophotometer. The total flavonoid content results were expressed by using rutin as the standard (mg of RE/g).

### Antioxidant activity

#### DPPH scavenging activity assay

The DPPH assay was selected to determine the free radical scavenging capacity of the sample according to [11] with slight modifications. The assay was performed in a 96-well microplate. In total, 100 μL of the samples with the concentration of 0.3 mg/mL were added to 100 μL of 1mM DPPH in ethanol. The mixture was well mixed and then placed in the dark environment to incubate for 30 min. Afterwards, the absorbances of the samples at 517 nm were measured using a microplate reader. The scavenging activity was expressed by the DPPH free radical inhibition rate in the samples with same concentration, which was calculated via the following formula:

$$\text{DPPH}\;\text{scavenging}\;\text{activity}(\%)=\left[\text{1}-\frac{\left({\text{A}}_{\text{s}}-{\text{A}}_{\text{c}}\right)}{{\text{A}}_{\text{0}}}\right]\times 100$$

where A_S_ refers to the absorbance of the sample with DPPH, A_C_ refers to the absorbance of the sample with ethanol, and A_0_ refers to the absorbance of DPPH with ethanol. All analyses were performed in triplicate.

#### ABTS scavenging activity assay

The solution with ABTS radical cations (ABTS^+^) was obtained via a reaction between an ABTS stock solution and an oxidizing solution. Then the solution was stored in the dark at room temperature for 12–16 h before use. Then, the ABTS radical cation solution was diluted with 80% ethanol until the absorbance at 734 nm reached to 0.7±0.05. A mixture of 10 μL samples with 200 μL of the ABTS diluted solution was kept in dark for 2–6 min at ambient temperature. Finally, the antioxidant capacity was measured using the absorbance at 734 nm. The antioxidant capacity was indicated by the Trolox Equivalent Antioxidant Capacity (TEAC). A calibration curve for Trolox was prepared at concentrations of 0.15, 0.3, 0.6, 0.9, 1.2 and 1.5 mM.

#### FRAP scavenging activity assay

The Ferric Reducing Ability of Plasma (FRAP) is a method for detecting the total antioxidant capacity. A FRAP working solution was produced by adding a buffer solution after sufficient mixing of a diluted tripyridyltriazine (TPTZ) solution. The working solution was incubated at 37 °C and the reactions should be completed within 1–2 hours. A mixture of 5 μL of the samples, 5 μL of deionized water and 180 μL of the FRAP solution was kept for 3–5 min at 37 °C. Then the antioxidant capacity was measured using the absorbance at 593 nm. The antioxidant capacity was indicated by the concentration of a FeSO_4_ standard solution. The calibration curve of FeSO_4_ was prepared at concentrations of 0.15, 0.3, 0.6, 0.9, 1.2 and 1.5 mM.

### α-Glucosidase inhibition

The α-glucosidase inhibition was evaluated using a spectrophotometric method according to Yang [25]. The assay was performed in a 96-well microplate. Then, 20-μL samples with a concentration of 2 mg/mL were mixed with 20 μL of α-glucosidase dissolved in PBS (phosphate buffer solution, 0.08 U/mL) and 40 μL of PBS (pH = 6.5). Afterwards, the mixture was kept at 37 °C for 10 min. Then, 40 μL of PNPG (0.375 mM) was mixed with the previous mixture and kept at 37 °C. After 30 mins, 80 μL of Na_2_CO_3_ (1M) was added to stop the reaction. The released 4-nitrophenol was measured by detecting the absorbance at 405 nm using a microplate reader. The α-glucosidase inhibition activity was expressed by the inhibition rate of the samples with the same concentration. The inhibition rate of the α-glucosidase activity was calculated via the following formula:

$$\text{inhibition(\%)}=\left[\text{1}-\frac{{\text{A}}_{\text{a}}-{\text{A}}_{\text{b}}}{{\text{A}}_{\text{c}}-{\text{A}}_{\text{d}}}\right]\times 100$$

in which Aa is the absorbance of the mixture with the samples and enzyme, Ab is the absorbance of mixture with the samples but without enzyme, Ac is the absorbance of the mixture with enzyme but without samples, and Ad is the absorbance of the mixture without samples or enzyme. The insufficient volume was supplemented with PBS.

### HPLC-ESI-MS/MS determination of alkaloid and flavonoid compounds

Mulberry leaf extracts were filtered through a 0.22-μm pore size membrane filter before injection. Mulberry leaf extracts were filtered through a 0.22-μm pore size membrane filter before injection. Mulberry leaf extracts were filtered through a 0.22-μm pore size membrane filter before injection. The HPLC-MS/MS consisted of a liquid chromatograph (Thermo, USA) and a mass spectrometer (Thermo, USA). The HPLC system is composed of a 1250 pump, an autosampler and a PDA detector.

#### HPLC-MS/MS determination of the alkaloids

The MS/MS parameters (collision energy, capillary temperature, etc.) were optimized with standard DNJ and fagomine under positive ion electrospray ionization. The electrospray ionization mass spectrometry detection was performed using the following optimized parameters shown in Table 2.

**Table 2:** Main compounds identified in mulberry leaves extracted by LC-ESI-MS/MS including molecular formula (MF), retention time(RT), LC- MS/MS parameters and MRM transitions.

Peak	Compounds	RT (min)	MF	Spray voltage(v)	Collision energy(v)	Tube lens	Quantitative transition(m/z)
1	fagomine	6.7	C_6_H_13_NO_3_	3000	19	54	147.55 > 129.76
2	DNJ	7.7	C_6_H_13_NO_4_	3000	16	54	164.14 > 145.87
3	CA	3.0	C_16_H_18_O_9_	3000	22	-42	352.75 > 199.70
4	isoquercitrin	20.6	C_21_H_20_O_12_	3000	32	-84	462.86 > 299.75
5	rutin	22.0	C_27_H_30_O_16_	3000	58	-112	608.63 > 299.60
6	quercetin	26.4	C_15_H_10_O_7_	3000	33	-67	300.74 > 150.87
7	kaempferide	27.6	C_16_H_12_O_6_	2500	33	-74	298.82 > 283.79

The mulberry leaf extracts (each 5 μL) were separated in an LC column (TSKgel Amide-80 5 μL, 2.0*150 mm, Tosoh Corporation, Tokyo, Japan). The LC mobile phases consisted of two solvents: mobile phase A, acetonitrile containing 0.1% formic acid; mobile phase B, deionized water containing 0.1% formic acid. Alkaloid compounds were eluted under the following conditions: 0.5 mL/min flow rate with a temperature of 40°C. The gradient profile was as follows: 0–1 min, 80%-61% A; 1–4.5 min, 61% A; 4.5–4.6 min, 61%-80% A and 4.6–13 min, 80% A. DNJ and fagomine were detected individually via MS/MS with multiple reaction monitoring (MRM) for transition of the parent ions to the product ions, which had been previously optimized. The concentrations of alkaloids in the mulberry leaves were calculated using calibration curves of the standard DNJ and fagomine.

#### HPLC-MS/MS determination of the phenols

Electrospray ionization mass spectrometry was performed in negative ion mode using the following optimized parameters shown in Table 2. The column used for separating flavonoids was a Thermo reserved phase C-18 column (50×2.1 mm, 1.9 μm, Thermo, USA). The LC mobile phases consisted of two solvents: mobile phase A, acetonitrile and mobile phase B, deionized water containing 0.2% formic acid. The gradient profile was as follows: 0–5 min, 5–8% A; 5–24 min, 8% A; 24–27 min, 8–55% A and 27–29 min, 55–90% A; 29-32min, 90–5% A. The column temperature was maintained at 25 °C with 2 μL sample each time at a flow rate of 0.5 mL/min.

### Statistical evaluation

Data on the contents of active ingredient, TPC, TFC, antioxidant activity and glycosidase inhibitory activity were analyzed variance (ANOVA). The results were expressed as means ± S.D. The significant difference was analyzed by Duncan test (P < 0.05) using SPSS 22.0 software (IBM, USA) to evaluate the differences among the samples from different areas.

## Results and discussion

### HPLC-ESI-MS/MS determination

In this study, the contents of the main alkaloids and phenols in mulberry leaves were analyzed. A new method, which can quantify 5 phenol compounds simultaneously in 32 minutes, was developed using a reversed-phase C-18 column (50×2.1 mm, 1.9 μm, Thermo, USA). The method used in a previous study could only quantify 2–3 components simultaneously, which was less effective than our method [26]. However, DNJ and fagomine are alkaloids with a higher polarity, which leads to an extremely short retention time of these two compounds in a C-18 chromatographic column. Therefore, the determination of DNJ and fagomine need a derivatization step with 9-fluorenylmethyl chloroformate (FMOC-Cl) [27]. To prevent instability with this complicated method, a TSKgel Amide-80 chromatographic column (5 μL, 2.0×150 mm) was selected to quantify DNJ and fagomine without derivatization. These 2 compounds can be quantified in 13 minutes using the new method. Based on the responsivity of the compounds, a negative mode and positive mode were selected for the quantification of phenols and alkaloids, respectively.

**Fig 1:**
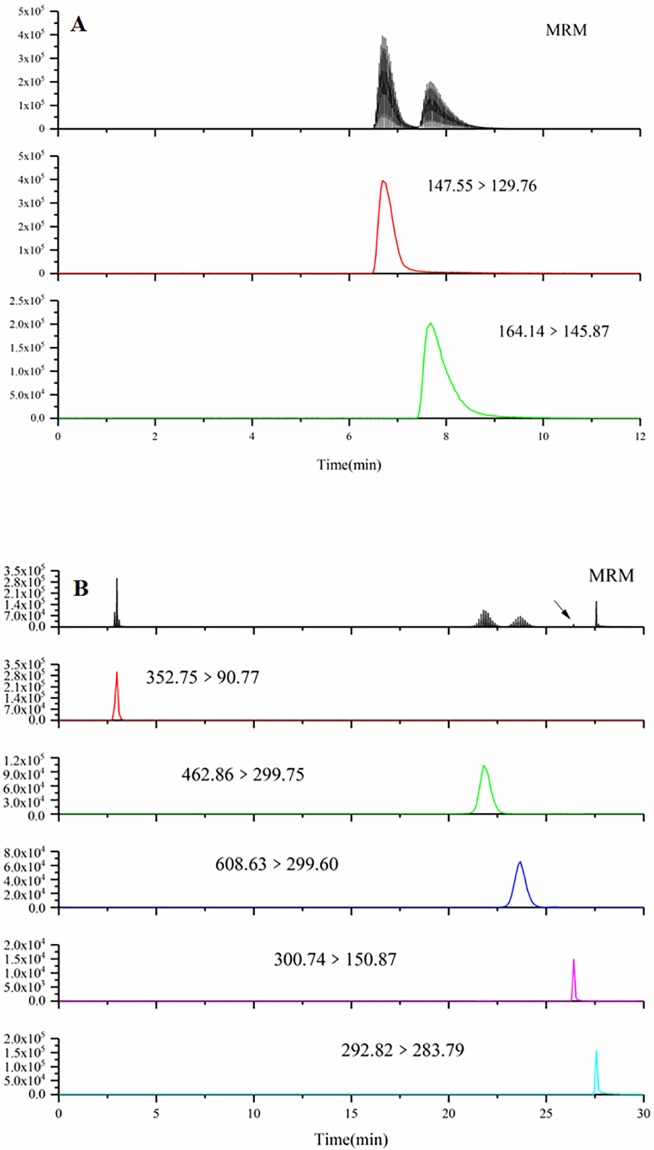
HPLC-ESI-MS/MS MRM chromatograms of alkaloid (A) and phenol compounds (B) identified in the samples. (TIF) (A) The red line represents the chromatogram of DNJ and the green line represents the chromatogram of fagomine. (B) The red, green, dark blue, pink, light blue line in represent the chromatograms of chlorogenic acid, isoquercitrin, rutin, quercetin and kaempferide, respectively.

The generated spectra of all compounds detected in the samples are shown in Fig 1 and S2 Fig. All the compounds were identified by comparing both retention times and MS spectral data (shown in Table 2) from the mulberry leaf samples and standards. Chromatographic peaks of all the compounds were separated, and the peak shapes presented well.

### Active compound content analysis

#### Content analysis of alkaloids

**Fig 2:**
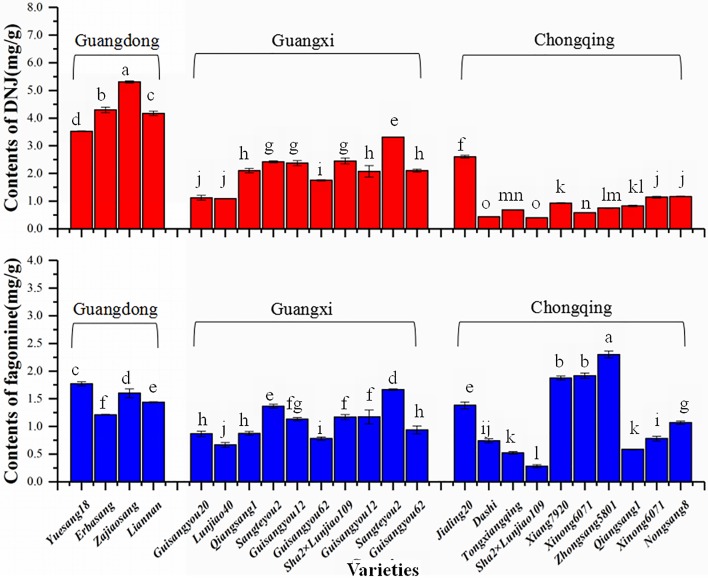
Contents of DNJ and fagomine from different collection areas. (TIF) The red columns mean the contents of DNJ from different collection areas and the blue columns mean the contents of fagomine.

DNJ and fagomine are the main alkaloid compounds in mulberry leaves, which were presented in all the samples. As shown in Table 3, sample 3 (Guangdong) had the highest DNJ content of 5.309±0.036 mg/g, whereas sample 21 (Chongqing) had the highest fagomine content of 2.300±0.060 mg/g, which were both obviously higher than those in other samples. The lowest contents of fagomine and DNJ were both detected in sample 18 (Chongqing) at 0.279±0.031 and 0.401±0.003 mg/g, respectively. Fig 2 shows the content comparison between DNJ and fagomine in all the samples. The DNJ contents of the samples from different areas exhibited a trend as follows: Guangdong > Guangxi > Chongqing. Moreover, the trends for temperature and precipitation in these areas were consistent with the trends of the contents of DNJ (Table 1): higher temperature and precipitation lead to higher DNJ contents. Thus, temperature and precipitation had positive correlations with the content of DNJ, and this result was also validated in previous studies [28].

**Table 3:** The content of the active compounds.

Sample No.	Alkaloids(mg/g)		Phenols(mg/g)				
	DNJ	Fagomine	Chlorogenic acid	Isoquercitrin	Rutin	Kaempferide	Quercetin
1	3.525±0.015^d^	1.771±0.035^c^	5.857±0.177^f^	1.204±0.036^k^^l^	1.878±0.003^h^	——^s^	ND
2	4.295±0.099^b^	1.211±0.007^f^	6.746±0.262^e^	2.133±0.110^f^	2.039±0.083^g^	——	ND
3	5.309±0.036^a^	1.600±0.078^d^	6.570±0.362^e^	0.537±0.034^r^	1.429±0.069^k^^l^	——	ND
4	4.173±0.077^c^	1.437±0.009^e^	5.417±0.070^g^^h^^i^	0.830±0.023^p^^q^	1.757±0.045^i^	——	——
5	1.121±0.086^j^	0.868±0.048^h^	4.476±0.194^j^	2.246±0.060^e^^f^	1.762±0.047^i^	——	——
6	1.092±0.009^j^	0.667±0.043^j^	3.510±0.139^k^	1.157±0.005^l^^m^	1.250±0.018^m^	——	——
7	2.102±0.082^h^	0.874±0.035^h^	6.646±0.263^e^	0.769±0.013^p^^q^	1.352±0.060^l^	——	ND
8	2.420±0.037^g^	1.366±0.035^e^	3.104±0.190^l^	1.005±0.050^n^^o^	1.870±0.096^h^	——	——
9	2.376±0.091^g^	1.131±0.027^f^^g^	6.721±0.26^1^^e^	1.621±0.024^i^	2.073±0.061^f^^g^	——	——
10	1.754±0.023^i^	0.781±0.029^i^	7.468±0.395^d^	1.704±0.052^h^^i^	2.755±0.083^b^	——	——
11	2.449±0.106^g^	1.168±0.045^f^	8.017±0.374^c^	1.841±0.041^g^	2.233±0.122^e^	——	ND
12	2.077±0.207^h^	1.171±0.125^f^	5.115±0.306^i^	1.053±0.026^m^^n^	2.139±0.034^e^^f^	——	ND
13	3.316±0.002^e^	1.665±0.014^d^	5.175±0.046^h^^i^	1.338±0.068^j^	2.335±0.046^d^	——	ND
14	2.100±0.055^h^	0.935±0.073^h^	6.897±0.044^e^	2.174±0.076^f^	2.699±0.053^b^^c^	——	ND
15	2.603±0.051^f^	1.382±0.062^e^	8.273±0.166^b^^c^	0.906±0.020^o^^p^	1.478±0.004^j^^k^	——	——
16	0.431±0.003^o^	0.740±0.038^i^^j^	8.607±0.291^b^	4.498±0.121^a^	1.725±0.042^i^	——	——
17	0.685±0.008^m^^n^	0.523±0.023^k^	5.260±0.089^h^^i^	3.165±0.097^c^	1.688±0.028^i^	——	ND
18	0.401±0.003^o^	0.279±0.031^l^	10.050±0.143^a^	3.430±0.044^b^	3.489±0.059^a^	——	ND
19	0.928±0.019^k^	1.875±0.035^b^	6.822±0.187^e^	3.148±0.039^c^	1.660±0.029^i^	——	ND
20	0.583±0.011^n^	1.917±0.048^b^	5.818±0.304^f^^g^	2.425±0.094^d^	0.960±0.029^n^	——	——
21	0.750±0.010^l^^m^	2.300±0.060^a^	5.573±0.02^f^^g^^h^	1.751±0.047^g^^h^	1.333±0.022^l^^m^	——	ND
22	0.827±0.029^k^^l^	0.585±0.006^k^	4.094±0.090^j^	1.371±0.046^j^	0.970±0.021^n^	——	ND
23	1.143±0.031^j^	0.780±0.042^i^	8.204±0.453^b^^c^	2.349±0.172^d^^e^	1.539±0.054^j^	——	——
24	1.165±0.012^j^	1.068±0.029^g^	8.044±0.173^c^	1.284±0.055^j^^k^	2.623±0.096^c^	——	——

^a-r^ means significantly different (P<0.05) in Duncan Test.

^s^ means the substance of the active compounds can be detected but cannot be quantified.

ND means the active compounds can not be detected.

For fagomine, samples 19–21 (Chongqing, Fuling) had highest content of fagomine compared with samples from the other sampling sites in Chongqing. Although Fuling did not have a precipitation as high as that of Guangdong and Guangxi, it is located at the confluence of the Yangtze River and Wujiang, where there are abundant water resources. The abundant water resource could probably promote the synthesis of fagomine. According to another study, fagomine was also found in the roots of *Xanthocersis zambesiaca* at a content 1.30 mg/g [29]; however, the contents of fagomine from Chongqing Fuling could reach to 2.30 mg/g, which were much higher than that of *Xanthocersis zambesiaca*. In addition, the biomass of the leaves was much higher than that of roots and leaves that were much easier to obtain, thus, mulberry leaves are more suitable as a resource of fagomine. Additionally, fagomine itself has a good hypoglycemic activity. Therefore, samples from Chongqing Fuling and Guangdong are suitable for producting healthy foods.

#### Content analysis of phenols

**Fig 3:**
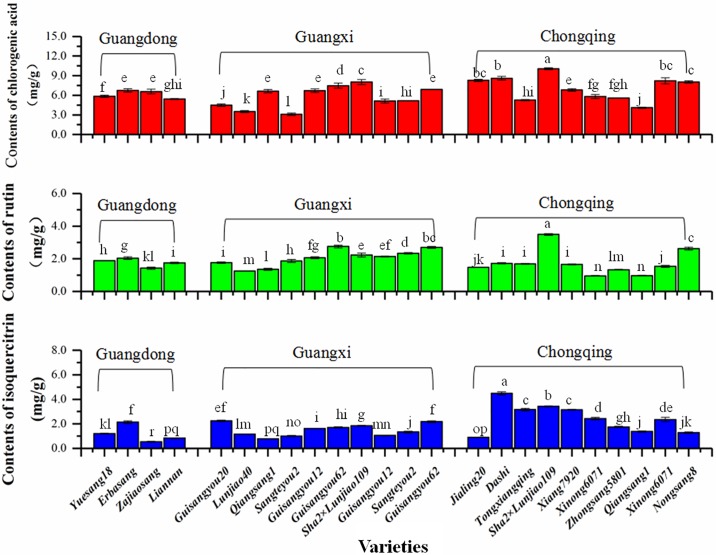
Contents of phenols from different collection areas. (TIF) The red columns mean the contents of chlorogenic acid from different collection areas, the green columns mean the contents of rutin and the blue columns mean the contents of isoquercitr.

Phenols are the main antioxidant constituents in mulberry leaves, and are associated with anti-inflammatory, antitumor and antibacterial activity[18, 30]. Five phenols (quercetin, isoquercitrin, rutin, chlorogenic acid, kaempferide) were detected in our study. However, due to their low content, the compunds quercetin and kaempferide could not be quantified (Table 3) in the sample extracts. Chlorogenic acid (CA) is a phenolic acid with the highest content among the five constituents. As shown in Fig 3 and Table 3, the content of chlorogenic acid (CA) ranged from 3.104–10.050 mg/g, and sample 18 (Chongqing) had the highest content. As shown in Fig 3, the CA content of Chongqing was slightly higher than that of Guangdong and Guangxi. Based on a previous study, *Lonicera japonica* is a plant rich in chlorogenic acid (4.18–4.92 mg/g) [31]. However, the CA contents in our samples were higher than that of *Lonicera japonica* which can be up to 10.05 mg/g. Additionally, mulberry trees are more widely planted than *Lonicera japonica* in China; thus, mulberry leaves, espicially from Chongqing, are more suitable at the potential resources of chlorogenic acid.

The flavonoids in mulberry leaves were also determined. The main flavonoids in mulberry leaves are isoquercitrin and rutin; however, their contents are not so high as CA. As shown in Fig 3 and Table 3, the content of isoquercitrin exhibited an increasing trend in the following order: Guangdong < Guangxi < Chongqing, ranging from 0.537–4.498 mg/g. This could be because the high latitude environment with lower temperature was more fit for the formation of isoquercitrin. Based on previous literatures, phenylalanine ammonia-lyase (PAL) is the first key enzyme that can be induced by environmental factors, and it is involved in the formation of flavonoids in the bio-synthesis pathway of mulberry leaves. At low temperatures, the expression of the PAL gene can be induced, which results in an increasing content of flavonoids [32]. The contents of rutin were not significantly different, which ranged from 0.960–3.489 mg/g, in which the content of sample 18 (Chongqing) had the highest content.

### TPC and TFC analysis

A previous study reported that mulberry leaf had both outstanding hypoglycemic activity [33] and good antioxidant activity due to the functional components contained in it. Therefore, the following analyses were selected to evaluate the health benefits of the mulberry leaf extracts from different growing areas: total phenolic content (TPC), total flavonoid content (TFC) analysis, antioxidant activity analysis and glycosidase inhibitory activity analysis.

Phenolic and flavonoid compounds are the major constituents that play an important role in the antioxidant activity of mulberry leaf extracts [11]. Therefore, the evaluation of TPC and TFC in mulberry leaf extracts is crucial for the determination of its antioxidant capacity. The results of the total phenolic content (TPC, mg of GAE/g) are presented in Table 4. TPC of samples varied from 24.43 mg/g to 39.38 mg/g, in which the TPC of sample 16 (Chongqing) was the highest and the TPC of sample 15 (Chongqing) and sample 17 (Chongqing) were relatively lower. Generally, the TPC could be arranged in the following sequence roughly: Guangdong > Guangxi > Chongqing. Additionally, the TPC of Sha2×Lunjiao109, which were collected from different sites (Guangxi and Chongqing), were both relatively high. This was probably because the TPC of this variety was relatively high, and the effect of the variety was greater than that of the region.

**Table 4:** Origins and species of samples and the total phenolics, total flavonoids, antioxidant activity (DPPH, ABTS, FRAP assay) and glycosidase inhibitory rate (GI).

Sample No.	Origin	Species	Total phenolics (GAE mg/g)	Total flavonoids (RE mg/g)	DPPH (%)	ABTS (mmol/g)	FRAP (mmol Fe^2+^/g)	GI (%)
1	Guangdong, Qingyuan	Yuesang 11	33.87±0.66^b^^c^^d^^e^	54.67±5.44^d^^e^^f^^g^^h^	80.78±2.35^a^^b^	0.126±0.005^b^^c^^d^^e^	232.31±11.21^c^^d^^e^	44.81±10.32^g^^h^
2	Guangdong,Guangzhou	Erbasang	39.38±1.44^a^	66.73±7.08^a^^b^^c^^d^^e^^f^	78.87±2.07^a^^b^	0.124±0.012^b^^c^^d^^e^	275.24±3.56^a^^b^^c^	68.77±7.29^a^^b^^c^^d^^e^^f^
3	Guangdong,Guangzhou	Zajiaosang	34.00±3.44^a^^b^^c^^d^	61.65±7.70^a^^b^^c^^d^^e^^f^	69.38±1.96^c^	0.139±0.012^a^^b^^c^	237.74±10.20^b^^c^^d^^e^	73.80±13.28^a^^b^^c^^d^^e^
4	Guangdong,Guangzhou	Liannan	27.95±1.52^f^^g^^h^	56.97±5.06^c^^d^^e^^f^^g^	63.70±2.59^d^^e^	0.101±0.012^f^^g^	228.26±11.77^c^^d^^e^^f^	59.76±6.91^d^^e^^f^^g^
5	Guangxi,Laibin	Guisangyou 20	34.85±9.71^a^^b^^c^	56.03±9.85^d^^e^^f^^g^	67.65±1.41^c^^d^	0.144±0.002^a^^b^	307.98±105.50^a^	61.10±7.60^d^^e^^f^^g^
6	Guangxi,Laibin	Lunjiao 40	26.63±0.58^g^^h^	38.69±10.11^h^^i^	48.32±2.08^g^	0.124±0.002^b^^c^^d^^e^	133.88±5.26^h^	81.47±11.83^a^^b^^c^
7	Guangxi,Hechi	Qiangsang 1	29.62±1.47^c^^d^^e^^f^	55.92±7.66^d^^e^^f^^g^	63.25±2.63^e^	0.092±0.006^g^	231.59±16.00^c^^d^^e^	44.50±3.51^g^^h^
8	Guangxi,Hechi	Teyou 2	24.12±2.53^g^^h^	50.67±0.72^f^^g^^h^^i^	50.54±2.03^g^	0.069±0.006^i^	177.57±16.29^f^^g^^h^	53.73±2.04^f^^g^
9	Guangxi,Hechi	Guisangyou 12	28.67±1.71^e^^f^^g^^h^	61.70±0.46^a^^b^^c^^d^^e^^f^	76.86±2.38^b^	0.072±0.010^h^^i^	226.68±10.90^c^^d^^e^^f^	78.85±8.42^a^^b^^c^^d^
10	Guangxi,Hechi	Guisangyou 62	36.51±2.50^a^^b^	75.73±1.87^a^^b^	81.41±0.59^a^	0.100±0.013^f^^g^	284.82±11.87^a^^b^	69.44±16.42^a^^b^^c^^d^^e^^f^
11	Guangxi,Nanning	Sha 2×Lunjiao 109	38.56±4.38^a^^b^	73.29±2.87^a^^b^	76.51±2.79^b^	0.088±0.004^g^^h^	288.01±15.46^a^^b^	64.79±14.58^c^^d^^e^^f^
12	Guangxi,Nanning	Guisangyou 12	36.60±3.91^a^^b^	70.28±2.89^a^^b^^c^^d^	66.25±1.82^c^^d^^e^	0.131±0.017^b^^c^^d^	283.10±20.25^a^^b^	60.03±12.84^d^^e^^f^^g^
13	Guangxi,Nanning	Sangteyou 2	27.43±0.67^f^^g^^h^	59.87±4.06^b^^c^^d^^e^^f^	63.89±2.28^d^^e^	0.126±0.014^b^^c^^d^^e^	200.01±18.11^d^^e^^f^^g^	66.64±6.01^b^^c^^d^^e^^f^
14	Guangxi,Nanning	Guisangyou 62	36.81±1.02^a^^b^	76.42±2.28^a^	76.58±3.04^b^	0.137±0.007^a^^b^^c^^d^	299.07±5.58^a^	87.47±13.46^a^
15	Chongqing,Dianjiang	Jialing 20	23.43±1.78^h^	38.32±11.33^i^	57.36±2.05^f^	0.105±0.006^e^^f^^g^	200.81±11.41^d^^e^^f^^g^	58.50±5.16^e^^f^^g^
16	Chongqing,Dianjiang	Dashi	39.23±6.31^a^^b^	68.06±5.48^a^^b^^c^^d^^e^	79.47±2.50^a^^b^	0.152±0.009^a^	286.74±34.02^a^^b^	23.36±4.72^i^
17	Chongqing,Dianjiang	Tongxiangqing	24.83±3.02^g^^h^	41.21±12.61^g^^h^^i^	66.49±2.59^c^^d^^e^	0.134±0.014^a^^b^^c^^d^	186.35±9.60^e^^f^^g^	29.54±15.04^h^^i^
18	Chongqing,Dianjiang	Sha 2×Lunjiao 109	34.50±1.35^a^^b^^c^^d^	52.39±15.46^e^^f^^g^^h^^i^	78.32±1.95^a^^b^	0.153±0.006^a^	269.81±9.12^a^^b^^c^	14.23±6.30^i^
19	Chongqing,Fuling	Xiang7920	33.12±0.35^b^^c^^d^^e^	59.73±3.80^b^^c^^d^^e^^f^	69.59±2.59^c^	0.125±0.008^b^^c^^d^^e^	213.50±18.11^d^^e^^f^^g^	85.51±7.47^a^^b^
20	Chongqing,Fuling	Xinong6071	34.09±1.14^a^^b^^c^^d^	72.57±24.45^a^^b^^c^	67.69±1.93^c^^d^	0.116±0.015^d^^e^^f^	241.10±10.28^b^^c^^d^	23.53±9.43^i^
21	Chongqing,Fuling	Zhongsang5801	34.34±2.42^a^^b^^c^^d^	52.25±5.27^e^^f^^g^^h^^i^	63.24±1.20^e^	0.131±0.018^b^^c^^d^	229.40±4.36^c^^d^^e^	78.66±15.24^a^^b^^c^^d^
22	Chongqing,Qianjiang	Qiangsang 1	29.73±0.81^c^^d^^e^^f^	42.26±5.97^g^^h^^i^	43.57±0.87^h^	0.107±0.013^e^^f^^g^	174.69±12.43^g^^h^	73.37±11.65^a^^b^^c^^d^^e^^f^
23	Chongqing,Qianjiang	Xinong6071	29.51±2.97^c^^d^^e^^f^	51.33±1.29^f^^g^^h^^i^	66.54±3.58^c^^d^^e^	0.119±0.018^c^^d^^e^^f^	193.02±9.26^d^^e^^f^^g^	53.95±7.60^f^^g^
24	Chongqing,Qianjiang	Nongsang 8	34.77±1.58^a^^b^^c^^d^	56.68±3.73^c^^d^^e^^f^^g^	69.40±2.99^c^	0.126±0.008^b^^c^^d^^e^	228.08±33.14^c^^d^^e^^f^	31.48±8.12^h^^i^

each value is expressed as means ± S.D. (n = 3).

^a-i^ means significantly different (P<0.05) in Duncan Test.

The total flavonoid content results (TFC, mg of RE/g) are expressed by using rutin as the standard. The results showed that (Table 4) the highest and lowest TFC were observed from sample 14 (Guangxi) and sample 15 (Chongqing) at 76.42±2.28 and 38.32±11.33 mg/g respectively. The TFC could be arranged in the following general sequence: Guangxi > Guangdong > Chongqing.

### Antioxidant activity analysis

DPPH, ABTS and FRAP assays were selected to determine the antioxidant activity of the mulberry leaf extracts, and the results are shown in Table 4. For the DPPH assay, to measure the antioxidant capacity of different samples, mulberry leaves extracts of the same concentration (0.3 mg/mL) were used and compared via the free radical scavenging rate (DPPH%). Among the DPPH% results, that of sample 10 (Guangxi) was the highest, which reached 81.41±0.59% and those of sample 1 (Guagnzhou), sample 2 (Guangdong), sample 16 (Chongqing) and sample 18 (Chongqing) were relatively higher. The DPPH% of sample 6 (Guangxi), sample 8 (Guangxi) and sample 22 (Chongqing) were 48.32±2.08%, 50.54±2.03% and 43.57±0.87%, respectively, which were relatively lower than in other samples. Generally, the DPPH%s of samples from Guangdong were slightly higher than those of Guangxi and Chongqing.

For the ABTS assay, the highest and lowest ABTS+ scavenging activity was found for sample 18 (Chongqing) and sample 8 (Guangxi) at 0.152±0.006 and 0.068±0.006 mmol Trolox/g, respectively, which were similar with the results of the DPPH radical scavenging activity assay. The ABTS radical scavenging activity values could be arranged as follows: Chongqing > Guangdong > Guangxi and the trend was more obvious than that of DPPH.

For the FRAP assay, the final results are expressed using the concentration of a FeSO_4_ standard solution (mmol Fe^2+^/g). The FRAP values ranged from 133.88±5.26 (sample 6 from Guangxi) to 307.98±105.50 (sample 5 from Guangxi) mmol Fe^2+^/g. The FRAP values showed the following general trend: Guangxi > Guangdong > Chongqing, except for sample 6. It could be found that the samples from Guangdong and Guangxi showed better antioxidant properties generally except the ABTS assay. This was probably because the ABTS+ scavenging activity was related to the content of isoquercitrin, for the samples from Chongqing had obviously higher contents of isoquercitrin. Generally, the antioxidant activities for different regions showed the following trend: Guangxi > Guangdong > Chonging.

### Glycosidase inhibitory activity analysis

Mulberry leaf is famous for decreasing blood glucose; thus, it has been used as a functional or medical food to control blood glucose [34]. The glysosidase inhibitory (GI) rate results were shown in Table 2. The GI rates of the samples ranged from 14.23±7.90% (sample 18 from Chongqing) to 87.47±13.46% (sample 14 from Guangxi). Additionally, the GI rate of sample 6 (Guangxi), sample 9 (Guangxi), sample 19 (Chongqing), sample 21 (Chongqing) and sample 22 (Chongqing) were relatively higher at over 70%. Acarbose is a hypoglycemic drugs commonly used for therapeutic purposes. Previous literature reported that Acarbose has a common side-effect in that it can lead to gastrointestinal dysfunction. This is because Acarbose has α-amylase inhibition ability, which leads to the polysaccharides remaining in the gastrointestinal tract and producing large amount of gas because of glycolysis, whereas DNJ does not have α-amylase inhibition [35]. Moreover, Acarbose may cause acute liver injury, whereas DNJ can not be metabolized by the liver.

Comparing the samples from different regions, the GI rate of these regions showed the following trend: Guangdong > Guangxi > Chongqing, which was similar with the trend of the contents of alkaloids. It can be inferred that the GI rate has a positive correlation the contents of alkaloids. Thus, according to Table 4. samples from Guangdong have the best glycosidase inhibition activity. The samples from Guangxi showed lower glycosidase inhibition activities than Guangdong slightly and Chongqing exhibited the lowest glycosidase inhibition activity.

## Conclusions

Mulberry leaf is a traditional Chinese medicine, which contains a vatiety of active ingredients. Twenty-four mulberry leaf samples collected from 3 regions in southern China were evaluated. Seven main active ingredients (1-deoxynojirimycin, fagomine, chlorogenic acid, rutin and isoquercitrin) were detected and quantified using HPLC-MS/MS. Additionally, the total phenolic and total flavonoid contents, antioxidant potential and glycosidase inhibitory capacity (hypoglycemic activity) were tested using different assays. According to the results, Guangdong had higher content of DNJ and the highest content of fagomine than other collecting areas generally. Additionally, samples from Guangdong exhibited better glysosidase inhibitory activity than other areas, which meaned that samples from Guangdong could be future sources of natural hypoglycemic products. The antioxidant activity exhibited a tendency as follows: Guangxi > Guangdong > Chongqing. Thus, mulberry leaves from Guangdong and Guangxi could be future sources of antioxidant products. Additionally, CA had a high content in mulberry leaf, thus mulberry leaf was more suitable as potential resources of CA better than *Lonicera japonica*. Therefore, the results of our research will be meaningfull for the full use of mulberry leaves and we believe that mulberry leaf will have a broad application prospect in health products such as hypoglycemic food, antioxidant health products and anti-inflammatory health products.

## Supporting information

S1 Fig Different collection area of mulberry leaves in south China. (DOCX)

S2 Fig Mass spectrum of 5 phenol and 2 alkaloid compounds in negative ionization mode. (DOC)

## References

[1] Sugiyama M, Katsube T, Koyama A, Itamura H. Effect of solar radiation on the functional components of mulberry (Morus alba L.) leaves. J. Sci. Food Agr. 2016; 96(11): 3915–3921. 26756109 10.1002/jsfa.7614

[2] Andallu B, Shankaran M, Ullagaddi R, Iyer S. In vitro free radical scavenging and in vivo antioxidant potential of mulberry (Morus indica L.) leaves. J. Herb. Med. 2014; 4(1): 10–17.

[3] Shin SO, Seo HJ, Park H, Song HJ. Effects of mulberry leaf extract on blood glucose and serum lipid profiles in patients with type 2 diabetes mellitus: A systematic review. Eur. J. Integr. Med.2016; 8(5): 602–608.

[4] Sánchezsalcedo EM, Amorós A, Hernández F, Martínez JJ. Phytochemical properties of white (Morus alba) and black (Morus nigra) mulberry leaves, a new food supplement. J. Food Nutr. Res. 2017; 5(4): 253–261.

[5] Liu C, Xiang W, Yu Y, Shi ZQ, Huang XZ, Xu L. Comparative analysis of 1-deoxynojirimycin contribution degree to α-glucosidase inhibitory activity and physiological distribution in *Morus alba* L. Ind. Crop Prod. 2015; 70: 309–315.

[6] Riche DM, Riche KD, East HE, Barrett EK, May WL. Impact of mulberry leaf extract on type 2 diabetes (Mul-DM): A randomized, placebo-controlled pilot study. Complement Ther. Med. 2017; 32: 105–108. doi: 10.1016/j.ctim.2017.04.006 28619294 10.1016/j.ctim.2017.04.006

[7] Chen CC, Liu LK, Hsu JD, Huang HP, Yang MY, Wang CH. Mulberry extract inhibits the development of atherosclerosis in cholesterol-fed rabbits. Food Chem. 2005; 91(4): 601–607.

[8] Chan KC, Yang MY, Lin MC, Lee YJ, Chang WC, Wang CJ. Mulberry Leaf Extract Inhibits the Development of Atherosclerosis in Cholesterol-Fed Rabbits and in Cultured Aortic Vascular Smooth Muscle Cells. J. Agric. Food Chem. 2013; 61(11): 2780–2788. doi: 10.1021/jf305328d 23428158 10.1021/jf305328d

[9] Chang YC, Yang MY, Chen SC, Wang CJ. Mulberry leaf polyphenol extract improves obesity by inducing adipocyte apoptosis and inhibiting preadipocyte differentiation and hepatic lipogenesis. J. Funct. Foods.2016; 21: 249–262.

[10] Andallu B, Varadacharyulu NC. Antioxidant role of mulberry (Morus indica L. cv. Anantha) leaves in streptozotocin-diabetic rats. Clin. Chim. Acta. 2003; 338(1–2): 3–10. 14637259 10.1016/s0009-8981(03)00322-x

[11] Bae SH, Suh HJ. Antioxidant activities of five different mulberry cultivars in Korea. LWT-Food Sci. Technol. 2007; 40(6): 955–962.

[12] Yogisha S, Raveesha KA. In-vitro antibacterial effect of selected medicinal plant extracts. J. Nat. Prod. 2009; 2: 64–69.

[13] Wang F, Li JR, Jiang YM. Polysaccharides from mulberry leaf in relation to their antioxidant activity and antibacterial ability. J. Food Process Eng. 2010; 33(1): 39–50.

[14] Yang NC, Jhoh KY, Tseng CY. Antihypertensive effect of mulberry leaf aqueous extract containing γ-aminobutyric acid in spontaneously hypertensive rats. Food Chem. 2012; 132(4): 1796–1801.

[15] Chen PN, Chu SC, Chiou HL, Kuo WH, Chiang CL, Hsieh YS. Mulberry anthocyanins, cyanidin 3-rutinoside and cyanidin 3-glucoside, exhibited an inhibitory effect on the migration and invasion of a human lung cancer cell line. Cancer Lett. 2006; 235(2): 248–259. doi: 10.1016/j.canlet.2005.04.033 15975709 10.1016/j.canlet.2005.04.033

[16] Masoodsadiq B, Akmal N, Mtauseef S, Karin S. Morus alba L. nature’s functional tonic. Trends Food Sci. Tech. 2008; 19(10): 505–512.

[17] Han SY. Mulberry resources and its diverse utilization. Guizhou Agr. Sci. 2006; 34(3): 118–121.

[18] Hu XQ, Jiang L, Zhang JG, Deng W, Wang HL, Wei ZJ. Quantitative determination of 1-deoxynojirimycin in mulberry leaves from 132 varieties. Ind. Crop Prod. 2013; 49(8): 782–784.

[19] Kimura T, Nakagawa K, Kubota H, Kojima Y, Goto Y, Yamagishi K, et al Food-grade mulberry powder enriched with 1-deoxynojirimycin suppresses the elevation of postprandial blood glucose in humans. J. Agric. Food Chem. 2007; 55(14): 5869–5874. doi: 10.1021/jf062680g 17555327 10.1021/jf062680g

[20] Taniguchi S, Asano N, Tomino F, Miwa I. Potentiation of glucose-induced insulin secretion by fagomine, a pseudo-sugar isolated from mulberry leaves. Horm. Metab. Res. 1998; 30(11): 679–683. doi: 10.1055/s-2007-978957 9918385 10.1055/s-2007-978957

[21] Jia Z, Tang M, Wu J. The determination of flavonoid contents in mulberry and their scavenging effects on superoxide radicals. Food Chem. 1999; 64(4): 555–559.

[22] Sato Y, Itagaki S, Kurokawa T, Ogura J, Kobayashi M, Hirano T, et al In vitro and in vivo antioxidant properties of chlorogenic acid and caffeic acid. Int. J. Pharmaceut. 2011; 403(1–2): 135–138. 10.1016/j.ijpharm.2010.09.035 20933071

[23] Zhang DY, Luo M, Wang W, Zhao CJ, Gu CB, Zu YG, et al Variation of active constituents and antioxidant activity in pyrola [*P*. *incarnata Fisch*.] from different sites in Northeast China. Food Chem. 2013; 141(3): 2213–2219. doi: 10.1016/j.foodchem.2013.05.045 23870950 10.1016/j.foodchem.2013.05.045

[24] Liu L, Pan YL. Analysis of the total flavonoid content in folium mori from different species. Chin. Arg. Sci. Bull. 2008; 24: 488–491.

[25] Yang Z, Wang Y, Wang Y, Zhang Y. Bioassay-guided screening and isolation of a-glucosidase and tyrosinase inhibitors from leaves of Morus alba. Food Chem. 2012; 131(2): 617–625.

[26] Ou-Yang Z, Cao X, Wei Y, Zhang WWQ, Zhao M, Duan JA. Pharmacokinetic study of rutin and quercetin in rats after oral administration of total flavones of mulberry leaf extract. Rev. Bras. Farmacogn. 2013; 23(5): 776–782.

[27] Kim JW, Kim SU, Lee HS, Kim I, Ahn MY, Ryu KS. Determination of 1-deoxynojirimycin in Morus alba L. leaves by derivatization with 9-fluorenylmethyl chloroformate followed by reversed-phase high-performance liquid chromatography. J. Chromatoqr. A. 2003; 1002(1): 93–99. 10.1016/s0021-9673(03)00728-3 12885082

[28] Yang B, Ou-Yang Z, Zhao M, Wu Y, Wang QQ. Dynamic study on the contents of 1-DNJ, rutin and polysaccharide in mulberry leaves in different growing seasons. J. Chin. Med. Mater. 2012; 35: 876–879.

[29] Amézqueta S, Galán E, Fuguet E, Carrascal M, Abián J, Torres JL. Determination of d-fagomine in buckwheat and mulberry by cation exchange HPLC/ESI-Q-MS. Anal. Bioanal. Chem. 2012; 402(5): 1953–1960. doi: 10.1007/s00216-011-5639-2 22207282 10.1007/s00216-011-5639-2

[30] Ahmad A, Gupta G, Afzal M, Kazmi I, Anwar F. Antiulcer and antioxidant activities of a new steroid from Morus alba. Life Sci. 2013; 92(3): 202–210. doi: 10.1016/j.lfs.2012.11.020 23270943 10.1016/j.lfs.2012.11.020

[31] Jia FQ, Yin NN, Lin L. Determination of Chlorogenic Acid in Honeysuckle Stem and Honeysuckle from Different Sources in Different Harvest Periods by HPLC. Qilu Pharm. Affair. 2007; 01.

[32] Yu XF, Li YZ, Zhang WWQ, Wang DJ, Wei Y, Ou-Yang Z. Correlation between Flavonoids, Activity of Phenylalanine Ammonia-lyase and Temperature in Mulberry Leaves around the Frost. Food Sci. http://www.spkx.net.cn/CN/ Accept 04 March 2016.

[33] Asano N, Yamashita T, Yasuda K, Ikeda K, Kizu H, Kameda Y, et al Polyhydroxylated Alkaloids Isolated from Mulberry Trees (Morus alba L.) and Silkworms (Bombyx mori L.). J. Agric. Food Chem. 2001; 49(9): 4208–4213. 11559112 10.1021/jf010567e

[34] Mudra M, Ercan-Fang N, Zhong L, Furne J, Levitt M. Influence of mulberry leaf extract on the blood glucose and breath hydrogen response to ingestion of 75 g sucrose by type 2 diabetic and control subjects. Diabetes care. 2007; 30(5): 1272–1274. doi: 10.2337/dc06-2120 17303787 10.2337/dc06-2120

[35] Chen XW, Han JQ. Clinical application and adverse reactions of acarbose. Chin. J. Pharmacoepidemiol. 2005; 14: 70–72.

