# Supplementary material for: Effect of different planting areas on the chemical compositions and hypoglycemic and antioxidant activities of mulberry leaf extracts in Southern China
Source: PLoS One. 2018 Jun 26;13(6):e0198072. doi: 10.1371/journal.pone.0198072 (PMC6019398; doi:10.1371/journal.pone.0198072)

**Supplementary data to:**

**Effect of different planting areas on the chemical compositions and hypoglycemic and antioxidant activities of mulberry leaf extracts in Southern China**

Jing-Yi Hao^123^, Yi Wan^123^, Xiao-Hui Yao^123^, Wei-Guo Zhao^123^, Run-Ze Hu^123^, Cong Chen^123^, Long Li^123*^, Dong-Yang Zhang^123*^

*1. College of Biotechnology and Sericultural Research Institute, Jiangsu University of Science and Technology, Zhenjiang,* *212018, P.R. China*

*2. Laboratory of Quality and Safty Risk Assessment for Sericulture Products and Edible Insect (Zhenjiang), Ministry of Agriculture, Zhenjiang, 212018, P.R. China*

*3. Quality Inspection Center for Sericultutal Products Ministry of Agriculture, Zhenjiang, 212018, P.R. China*

** Corresponding author. Tel./fax: +86 511 85616777.*

*E-mail address: lilong10029@126.com; zhangdongyang1987@126.com*

**Supporting information**

**Figure S1** Different collection area of mulberry leaves in south China. Guangdong, Guangzhou (A) Guangdong, Qingyuan (B) Guangxi, Nanning (C) Guangxi, Xincheng (D) Guangxi, Hechi (E) Chongqing, Qianjiang (F) Chongqing, Fuling (G) Chongqing, Dianjiang (H).


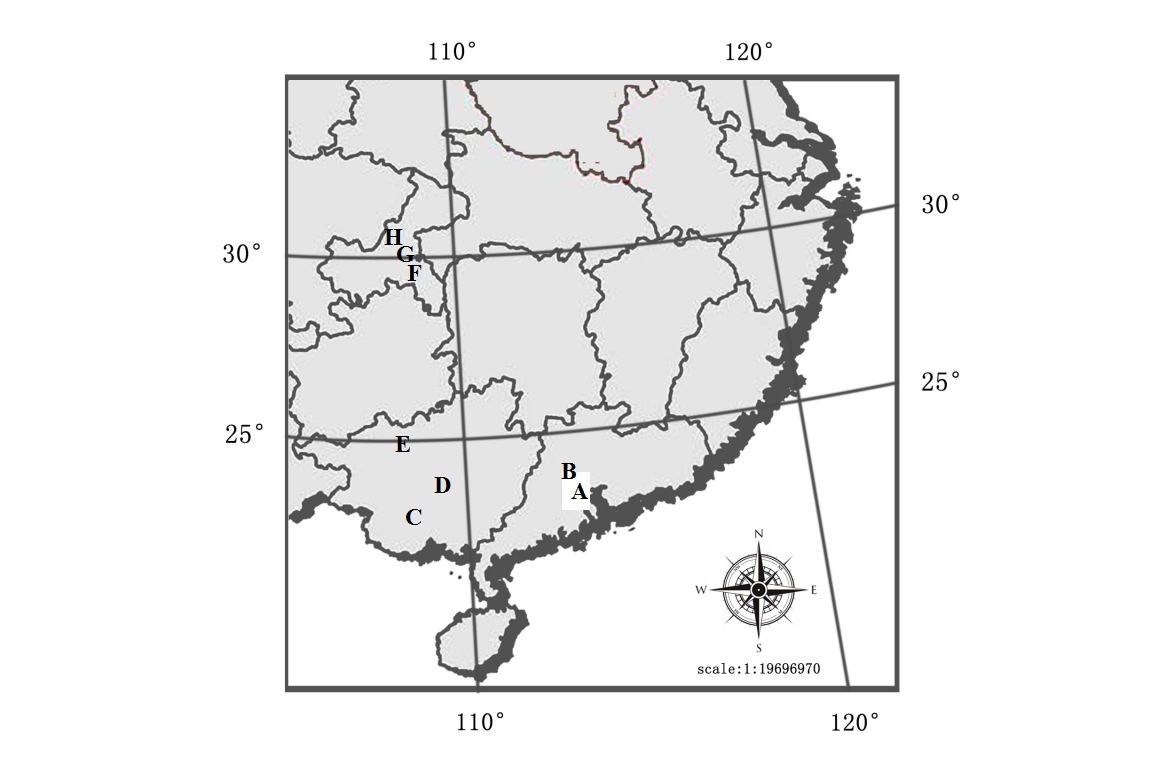

Supplement: S1 Fig — (DOCX) [file pone.0198072.s001.docx]
