# Supplementary material for: Effect of different planting areas on the chemical compositions and hypoglycemic and antioxidant activities of mulberry leaf extracts in Southern China
Source: PLoS One. 2018 Jun 26;13(6):e0198072. doi: 10.1371/journal.pone.0198072 (PMC6019398; doi:10.1371/journal.pone.0198072)

**Supplementary data to:**

**Effect of different planting areas on the chemical compositions and hypoglycemic and antioxidant activities of mulberry leaf extracts in Southern China**

Jing-Yi Hao^123^, Yi Wan^123^, Xiao-Hui Yao^123^, Wei-Guo Zhao^123^, Run-Ze Hu^123^, Cong Chen^123^, Long Li^123*^, Dong-Yang Zhang^123*^

*1. College of Biotechnology and Sericultural Research Institute, Jiangsu University of Science and Technology, Zhenjiang,* *212018, P.R. China*

*2. Laboratory of Quality and Safty Risk Assessment for Sericulture Products and Edible Insect (Zhenjiang), Ministry of Agriculture, Zhenjiang, 212018, P.R. China*

*3. Quality Inspection Center for Sericultutal Products Ministry of Agriculture, Zhenjiang, 212018, P.R. China*

** Corresponding author. Tel./fax: +86 511 85616777.*

*E-mail address: lilong10029@126.com; zhangdongyang1987@126.com*

**Supporting information**

**Figure S2** Mass spectrum of 5 phenol and 2 alkaloid compounds in negative ionization mode. Chlorogenic acid (A) Isoquercitrin (B) Rutin (C) Quercetin (D) Kaempferide (E) DNJ (F) Fagomine (G).


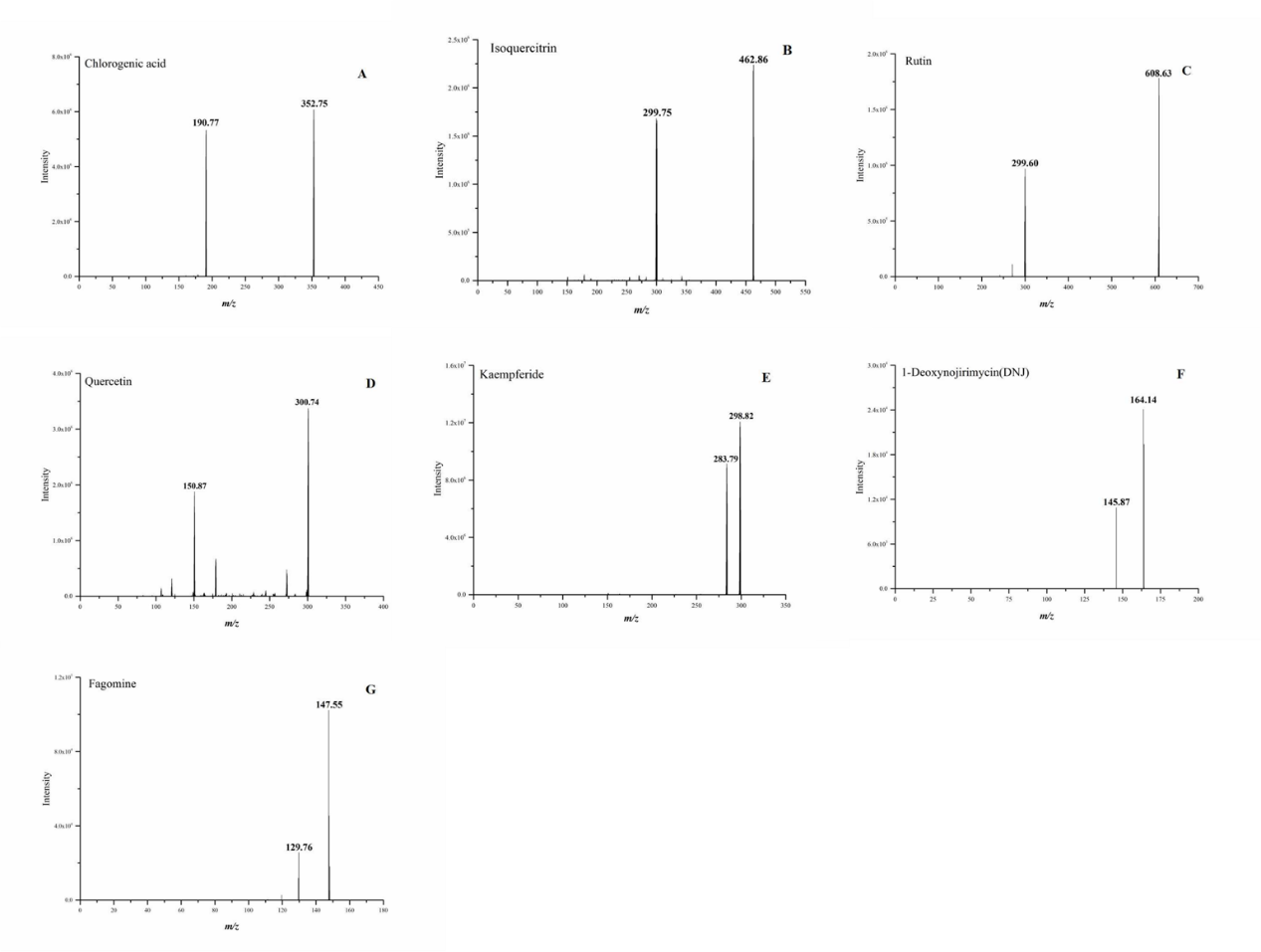

Supplement: S2 Fig — (DOC) [file pone.0198072.s002.doc]
